# Emergency department-based testing for xylazine and other novel psychoactive substances in Central Alabama: a feasibility study

**DOI:** 10.1186/s12954-026-01401-5

**Published:** 2026-01-17

**Authors:** William Bradford, Daniel Dye, Rebecca Jensen, Reed Bratches, Stacy Marshall, Ellen Eaton, Mary Figgatt, Whitney Taylor, Lauren A Walter, David Goodman-Meza, Stefan Kertesz, Karen S. Scott

**Affiliations:** 1 Division of Infectious Diseases, University of Alabama at Birmingham, Building 8th floor 1808 7th Ave S, Birmingham, AL 35233 USA; 2 Jefferson County Coroner/Medical Examiner’s Office, Birmingham, AL USA; 3 Department of Pathology, University of Alabama at Birmingham, Birmingham, AL USA; 4 School of Public Health, University of Alabama at Birmingham, Birmingham, AL USA; 5 School of Nursing, University of Alabama at Birmingham, Birmingham, AL USA; 6 Department of Emergency Medicine, University of Alabama at Birmingham, Birmingham, AL USA; 7 The Miriam Hospital, Brown University Health, Providence, RI USA; 8 Department of Medicine, Warren Alpert Medical School at Brown University, Providence, RI USA; 9 Kirby Institute, University of New South Wales Sydney, Sydney, Australia; 10 Birmingham Alabama Veterans Health Care System, Birmingham, AL USA; 11 Division of General Medicine and Population Health, University of Alabama at Birmingham, Birmingham, AL USA; 12 Division of Laboratory Medicine, University of Alabama at Birmingham, Birmingham, AL USA

**Keywords:** Xylazine, Fentanyl, Opioid, Cohort, Adulterants, Alabama

## Abstract

Alabama, like other states in the Deep South, lacks comprehensive testing for novel psychoactive substances (NPS) and adulterants like xylazine, leaving gaps in the detection of drug supply changes. From August 2024 to July 2025, we implemented an active testing approach at an emergency department (ED) in central Alabama among people with active illicit fentanyl use. Testing residual biological specimens collected as part of health care offers a potentially useful window into the prevalence of NPS in the drug supply, particularly in communities where traditional drug checking services might be impermissible or difficult to resource. In this study, we used liquid chromatography-quadrupole time of flight mass spectrometry (LC-QTOF-MS) to test the participants’ residual biological specimens (blood, urine) coupled with a survey focused on demographics and drug use. We enrolled 37 participants who completed surveys, and 31 who completed LC-QTOF-MS testing. 84% of participants were white (*n* = 31), 51.4% were male (*n* = 19), 54.1% were homeless (*n* = 20), and 45.9% resided in rural areas (*n* = 17). In participants with comprehensive toxicology testing (*n* = 31, 83.8%), we detected xylazine in 25 (80.6%). We identified the first confirmed case of medetomidine exposure in the state of Alabama. We also identified a high proportion of samples with the NPS *o*-methylfentanyl (*n* = 9, 29.0%), the first published identification of this substance in the region to our knowledge. Among participants with toxicology testing, 59.3% (*n* = 16) had previously heard of xylazine and 41.9% (*n* = 13) had knowingly used it in the past. This pilot study demonstrated the feasibility and practical utility of ED-based residual biological specimen illicit drug surveillance. This approach has the potential to identify new emerging substances in the unregulated drug supply.

**Supplementary Information:**

The online version contains supplementary material available at 10.1186/s12954-026-01401-5.

## Background

Drug supply changes, including the emergence of veterinary pharmaceuticals and novel psychoactive substances (NPS), are a defining characteristic of the contemporary drug use landscape, particularly for opioids [1–3]. Xylazine, a veterinary anesthetic, has been detected increasingly in the United States (US) drug supply. Xylazine use was associated with 10.9% of US illicit fentanyl deaths by June 2022, a 276% increase from 2019 [4–6]. First detected at high prevalence in the US Northeast, recent data has indicated that it has spread throughout the US, where it is nearly always found in combination with illicit fentanyl [2, 5, 7–10]. In addition to large, distinctive wounds [2, 11–16], xylazine is associated with other harms such as a unique withdrawal syndrome [17–19], heavy sedation [2, 20–22], and other effects [3, 20, 21, 23]. Based on these and other perceived harms, many people who use drugs (PWUD) report a preference to avoid xylazine [2, 20, 21]. Unfortunately, exposure to xylazine and other toxic additives appear to occur without the knowledge of PWUD despite their express preference to avoid such substances [16, 17, 20, 24]. 

Traditional drug supply surveillance approaches focused on identifying new additives face significant barriers in under-resourced areas, particularly the American Deep South, where drug checking and syringe service programs (SSPs) are less common due primarily to legal constraints [25, 26]. Furthermore, postmortem drug testing is severely limited by the reliance on coroners instead of medical examiners, especially in rural areas. These offices rarely have resources to outsource the type of high-resolution drug testing necessary to detect NPS as part of the medicolegal death investigation of overdose deaths [27, 28]. These limitations hamper postmortem toxicology surveillance and real-time monitoring of emerging NPS. In an analysis from Jefferson County, Alabama, which does rely on a medical examiner, xylazine was present in up to 39.3% of monthly drug overdose deaths between 2019 and 2023 and has been consistently present in decedents since 2021 [29]. Active surveillance systems enable timely, targeted responses to emerging drug supply changes and have been instrumental in the overdose response in Philadelphia and other impacted jurisdictions [24, 30]. Such systems are urgently needed in regions with restrictive policies, where access to traditional touchpoints for information and harm reduction resources are limited. The key challenge is configuring such a system to operate within the context of local legalities and resource constraints.

Residual biological specimen-based testing represents a legally viable surveillance approach that leverages specimens originally collected for clinical care but slated for discard after diagnostic testing is complete. Using this approach, leftover blood, urine, or other biological samples from emergency department (ED) patients and other acute care encounters are systematically analyzed for substances to identify changing drug exposure patterns. Residual biological specimen testing offers several features: it operates within existing healthcare workflows without requiring additional specimen collection, provides objective biochemical data rather than self-reported drug use information, and can detect substances that patients may be unaware they consumed, producing timely and high-level data at scale for use in epidemiologic studies [25, 31, 32]. 

On this background, we developed an ED-based active surveillance program to detect NPS and assess xylazine prevalence and user awareness in this population. We hypothesized that xylazine is prevalent and exposure occurs largely without the knowledge of PWUD, with risk factors for xylazine exposure similar to national patterns including homelessness and polysubstance use [4, 16, 18]. We furthermore hypothesized that a residual biological specimen-based surveillance approach would prove both feasible in this legally restrictive environment and effective for identifying emerging NPS.

## Methods

### Participant recruitment and selection

This prospective cohort study recruited participants from the University of Alabama at Birmingham (UAB) main ED, over a 1-year period from August 2024 to July 2025. Located in a medium-sized metropolitan area in the Southeastern US, UAB serves as a regional referral center and as the main safety net hospital for the region; the ED sees approximately 75,000 patients per year. Screening and enrollment were carried out during daytime hours Monday through Friday. Participants were approached by an ED-based recruitment team if they were age 18 or older and had a urine drug screen (UDS) result positive for fentanyl from their current ED encounter. Approaches were generally carried out Monday through Friday during business hours with a few exceptions. Prospective participants were eligible for enrollment if they self-reported use of non-prescribed opioids in the past 96 h. This relatively short 96-hour timeframe was chosen to increase the likelihood of identifying toxic substances with short half-lives. Exclusion criteria were incarceration, pregnancy, and inability to complete the study questionnaire in English. For those who agreed to participate, we offered a financial incentive of $20 via payment card (ClinCard®). Participants also had the option to receive the results of their xylazine testing with the understanding that the testing was for research rather than clinical purposes. When a positive result was delivered to a study participant, it was delivered via phone along with information on xylazine as well as harm reduction approaches to mitigate harms related to use of substances containing xylazine. All study activities were reviewed and approved by the UAB Institutional Review Board (IRB-300012605).

### Data collection

We administered a survey to participants via Qualtrics® (Provo, UT), in which we collected sociodemographic data, medical history, surgical history, and detailed substance use behaviors. We asked questions related to prior knowledge of and attitudes toward xylazine, including participant preferences for approaches to reduce the harms associated with xylazine exposure, using Likert scales. Prior to asking about attitudes toward harm reduction measures and use preferences, we provided all participants with a paragraph of education about what xylazine is and the harms associated with its use. During development, we piloted the survey among people with previous substance use as well as experts in survey-based data collection in PWUD. We incorporated their feedback and designed the instrument to take approximately 15 min to complete to accommodate completion in an ED setting.

In addition to administering the survey, study personnel requisitioned residual biological specimens including urine, whole blood, serum, and plasma (any sample available from the ED workup) from the lab, then aliquoted the specimens and stored them at −20 °C. In some cases, these specimens had already been discarded or were otherwise unsuitable for testing (e.g., if there was too little sample left). On an approximately bimonthly basis, study personnel sent samples to a reference lab, the Center for Forensic Science Research and Education (CFSRE; Horsham, PA), for liquid quadrupole time-of-flight mass spectrometry (LC-QTOF-MS) testing against a reference library of known and emerging drugs and drug additives. We also collected additional prospective and retrospective clinical information (mortality, overdose death, key comorbidities) via chart review. We used REDCap tools hosted at UAB to manage study data [33]. 

### Exposure and outcome definitions

We defined the primary exposure of xylazine as the presence of xylazine identified via LC-QTOF-MS in any blood, serum, urine, or plasma specimen. We defined homelessness using participants’ answers to the NIH Common Data Elements screener “Are you currently living in transitional housing, staying in a shelter, or experiencing homelessness?” We defined rurality as a locale identity of “Remote Rural,” “Fringe Rural,” or “Distant Rural” as specified by Geographic Information Systems mapping of participant-reported mailing addresses to National Center for Educational Statistics data [34]. Insurance status was based on the insurance recorded during ED encounter registration. We constructed all medical and surgical history variables by integrating participant-reported history with corresponding diagnoses identified in electronic health records (EHRs) to create a composite variable representing both EHR and self-reported history relevant to a condition. A lookback period of 1 year was used to identify diagnoses in the EHR. Substance use behaviors were collected via participant self-report through survey data (complete survey instrument available for download in supplemental materials). In the interest of comparing our results to other studies, we also compared participant-reported xylazine use with LC-QTOF-MS detection of xylazine. We used our LC-QTOF-MS data to infer the most likely parent compound (e.g., “norfentanyl” would be interpreted as a parent compound “fentanyl”) using the principle of parsimony and judgement of a medical and forensic toxicologist. Our outcomes of interest were 3-month mortality and 3-month readmission (defined as inpatient admission) as evidenced by review of the UAB health system records.

### Statistical analysis

We summarized variables using descriptive statistics with measures of central tendency (mean, median), dispersion (standard deviation, interquartile range), and distribution (frequency, percentage). For between-group differences, we used appropriate nonparametric tests as indicated (chi-square and Fisher’s Exact test for categorical variables; Kruskal–Wallis for continuous variables). We used nonparametric tests due to the small sample sizes and nonnormality of the data. Missing data were treated as missing at random and excluded from their respective analyses; bivariate comparisons were completed using listwise complete case analysis. We used ArcGIS Pro version 3.2.0 to carry out location-based analyses. We used SAS version 9.4 (Cary, NC) for all statistical analysis. We used RStudio version 2025.05.01 for Likert data visualization. We used Claude Opus 4.1 (San Francisco, CA) for statistical coding assistance and debugging.

## Results

### Cohort characteristics

A total of 37 people met inclusion criteria and were enrolled. Comprehensive toxicology testing results were available for 31; only these participants were used for the primary analysis (Tables 1 and 2). Of these participants, 54.8% were male (*n* = 17), 83.3% were white (*n* = 25), and 53.3% were homeless (*n* = 16). 39% (*n* = 12) were uninsured. Most participants for whom address data was available 51.9% (*n* = 14) were rural. The median distance in miles from the ED was 20.6 miles (range: 1.1–150.6). Wounds were prevalent (*n* = 13, 41.9%), as was anemia (*n* = 11, 35.5%), while other studied comorbidities (dysglycemia, compartment syndrome/rhabdomyolysis) were rare. Hepatitis C was prevalent (*n* = 17, 54.8%) and reported to have been treated in a minority of participants (*n* = 3, 18.8%). A total of 25 participants (80.6%) tested positive for xylazine by LC-QTOF-MS testing. Most participants reported fentanyl use (Table 2; *n* = 30, 96.8%) followed by heroin (*n* = 14, 45.2%). Most reported active injection drug use (*n* = 21, 67.7%), while almost all (*n* = 25, 80.6%) had used injection drugs at some point in the past. Stimulant and opioid co-injection was also common (*n* = 12, 38.7%). The most commonly co-used substances reported by participants were tobacco (*n* = 31, 100.0%), methamphetamine (*n* = 27, 87.1%), and cocaine (*n* = 11, 35.5%). Overall, inpatient admission within 3 months occurred in 2 participants (6.5%). No episodes of mortality within 3 months of study enrollment were identified by review of the EHR.

**Table 1:** Baseline characteristics and comorbidities by xylazine exposure status (*N* = 31)

Characteristic	Total (*N* = 31)	Xylazine positive (*n* = 25)	Xylazine negative (*n* = 6)	*p *value
Sociodemographics				
Age (mean (SD))	38.5 (9.7)	37.6 (9.7)	42.3 (9.2)	0.19
Sex, n (%)				0.37
Male	17 (54.8)	15 (60.0)	2 (33.3)	
Female	14 (45.2)	10 (40.0)	4 (66.7)	
Race, n (%)				1.00
White	25 (83.3)	20 (83.3)	5 (83.3)	
Black/African American	5 (16.7)	4 (16.7)	1 (16.7)	
Missing*	1	1	0	
Homeless, n (%)	16 (53.3)	12 (50.0)	4 (66.7)	0.66
Missing	1	1	0	
Insurance type, n (%)				
Private	9 (34.6)	8 (36.4)	1 (25.0)	1.00
Public	5 (19.2)	3 (13.6)	2 (50.0)	0.15
Uninsured	12 (46.2)	11 (50.0)	1 (25.0)	0.60
Missing	5	3	2	
Rural, n (%)	14 (51.9)	11 (52.4)	3 (50.0)	1.00
Missing	4	4	0	
Miles from home to ED (median (range))	20.6 (1.1-150.6)	20.6 (1.1-150.6)	18.4 (1.2–50.5)	0.68
Medical history				
Past year history, n (%)				
DVT/PE	3 (9.7)	3 (12.0)	0 (0.0)	1.00
Anemia	11 (35.5)	8 (32.0)	3 (50.0)	0.64
Dysglycemia	3 (9.7)	2 (8.0)	1 (16.7)	0.49
Compartment syndrome or rhabdomyolysis	1 (3.2)	1 (4.0)	0 (0.0)	1.00
Wounds	13 (41.9)	10 (40.0)	3 (50.0)	0.68
HCV diagnosis, n (%)	17 (54.8)	15 (60.0)	2 (33.3)	0.37
HCV treatment, n (%)	3 (18.8)	2 (33.3)	1 (50.0)	0.37
Missing	15	11	4	
HIV diagnosis, n (%)	2 (6.5)	1 (4.0)	1 (16.7)	0.35
Medical outcomes				
3-month admission, n (%)	2 (6.5)	2 (8.0)	0 (0.0)	1.00
3-month mortality, n (%)	0 (0.0)	0 (0.0)	0 (0.0)	N/A

*When present, missing values are reported in the table but were excluded from bivariate analyses

Abbreviations: DVT: Deep vein thrombosis; ED: Emergency department; HCV: Hepatitis C virus; HIV; Human immunodeficiency virus; PE: Pulmonary embolism; SD: Standard deviation

**Table 2:** Use behaviors and toxicology results by xylazine exposure status (*N* = 31)

Characteristic	Total (*N* = 31)	Xylazine positive (*n* = 25)	Xylazine negative (*n* = 6)	*p *value
Use behaviors				
IDU, n (%)				
Ever	25 (80.6)	19 (76.0)	6 (100.0)	0.31
Current	21 (67.7)	16 (64.0)	5 (83.3)	0.63
Intentional stimulant coinjection, n (%)	12 (54.5)	9 (56.3)	3 (50.0)	1.00
Missing*	9	9	0	
Any IDU-related risk behavior, n (%)	25 (100.0)	19 (100.0))	6 (100.0)	N/A
Missing	6	6	0	
Self-reported typical opioid use, n (%)				
Fentanyl	30 (96.8)	24 (96.0)	6 (100.0)	1.00
Heroin	14 (45.2)	6 (24.0)	1 (16.7)	1.00
Prescription pills	4 (12.9)	4 (16.0)	0 (0.0)	0.56
Non-prescription pills	7 (22.6)	6 (24.0)	1 (16.7)	1.00
Xylazine understanding				
Heard of xylazine, n (%)	16 (59.3)	15 (68.2)	1 (16.7)	0.13
Missing	4	3	0	
Reported use of xylazine, n (%)	13 (41.9)	12 (48.0)	1 (16.7)	0.36
Xylazine use preference, n (%)				
Very much prefer to avoid	24 (77.4)	19 (76.0)	5 (83.3)	1.00
Any other answer	7 (22.6)	6 (24.0)	1 (16.7)	1.00

*When present, missing values are reported in the table but were excluded from bivariate analyses

Abbreviations: IDU: Injection drug use

**Table 3:** Toxicology testing results by xylazine exposure status (*N* = 31)

Characteristic	Total (*N* = 31)	Xylazine positive (*n* = 25)	Xylazine negative (*n* = 6)	*p *value
Number of classes of substance detected (mean (SD))	3.7 (1.2)	3.9 (1.1)	2.8 (1.2)	0.06
Drug class detection, n (%)				
Barbiturates	0 (0.00)	0 (0.00)	0 (0.00)	N/A
Benzodiazepines	10 (32.3)	9 (32.0)	2 (33.3)	1.00
Buprenorphine	7 (22.6)	6 (24.0)	1 (16.7)	1.00
Cocaine	14 (45.2)	13 (52.0)	1 (16.7)	0.18
Fentanyl	31 (100.0)	25 (100.0)	6 (100.0)	N/A
Hydrocodone	1 (3.2)	1 (4.0)	0 (0.0)	1.00
Methadone	4 (12.9)	4 (16.0)	0 (0.0)	0.56
Methamphetamine	27 (87.1)	22 (88.0)	5 (83.3)	1.00
Oxycodone	1 (3.2)	0 (0.0)	1 (16.7)	0.19
Any MOUD agent	11 (35.5)	10 (40.0)	1 (16.7)	0.38
LC-QTOF-MS detection, n (%)				
Cutting agents	28 (90.3)	23 (92.0)	5 (83.3)	0.49
Fentanyl analogs	15 (48.4)	14 (56.0)	1 (16.7)	0.17
Psychiatric medications	10 (32.3)	8 (32.0)	2 (33.3)	1.00
Methcathinone	4 (12.9)	4 (16.0)	0 (0.0)	0.56
*O*-methylfentanyl	9 (29.0)	9 (36.0)	0 (0.0)	0.14

Abbreviations: LC-QTOF-MS: Liquid chromatography-quadrupole time of flight mass spectrometry

**Fig. 1:**
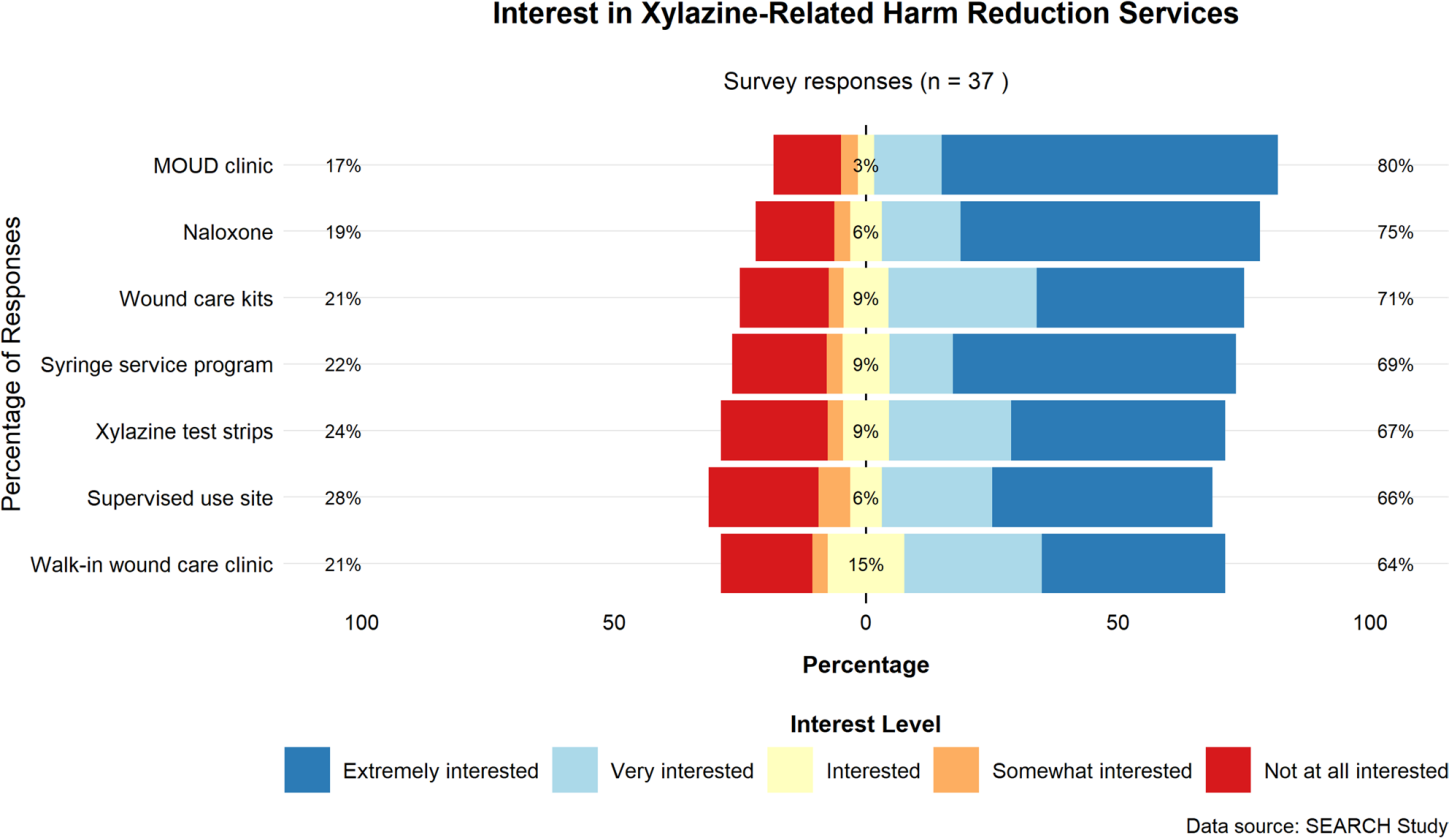
Harm reduction measure interest for the sample, measured on Likert scale from 1 (not at all interested) to 5 (extremely interested) (*N* = 37)

Among the queried treatment and service measures (Fig. 1), the more preferred interventions were access to a local free or low-cost clinic offering medication for opioid use disorder (MOUD; mean Likert score 4.2/5) and free or low-cost naloxone (4.0/5), while a supervised use site (3.6/5), wound care clinic (3.6/5), and xylazine test strips (3.6/5) were relatively less preferred. Among all study participants, there was an overwhelming strong preference to avoid xylazine exposure (Table 2; *n* = 24, 77.4%). Less than half of participants believed that they had been exposed to xylazine in the past (*n* = 13, 41.9%). The average study cost per participant was $595.73 USD, requiring an average of 12.9 h of research assistant time per participant. An average of 3.1 participants were enrolled per month across the entire study period. Median laboratory turnaround time was 55 days (range: 8–127 days) for comprehensive toxicology testing.

### Toxicology results

**Fig. 2:**
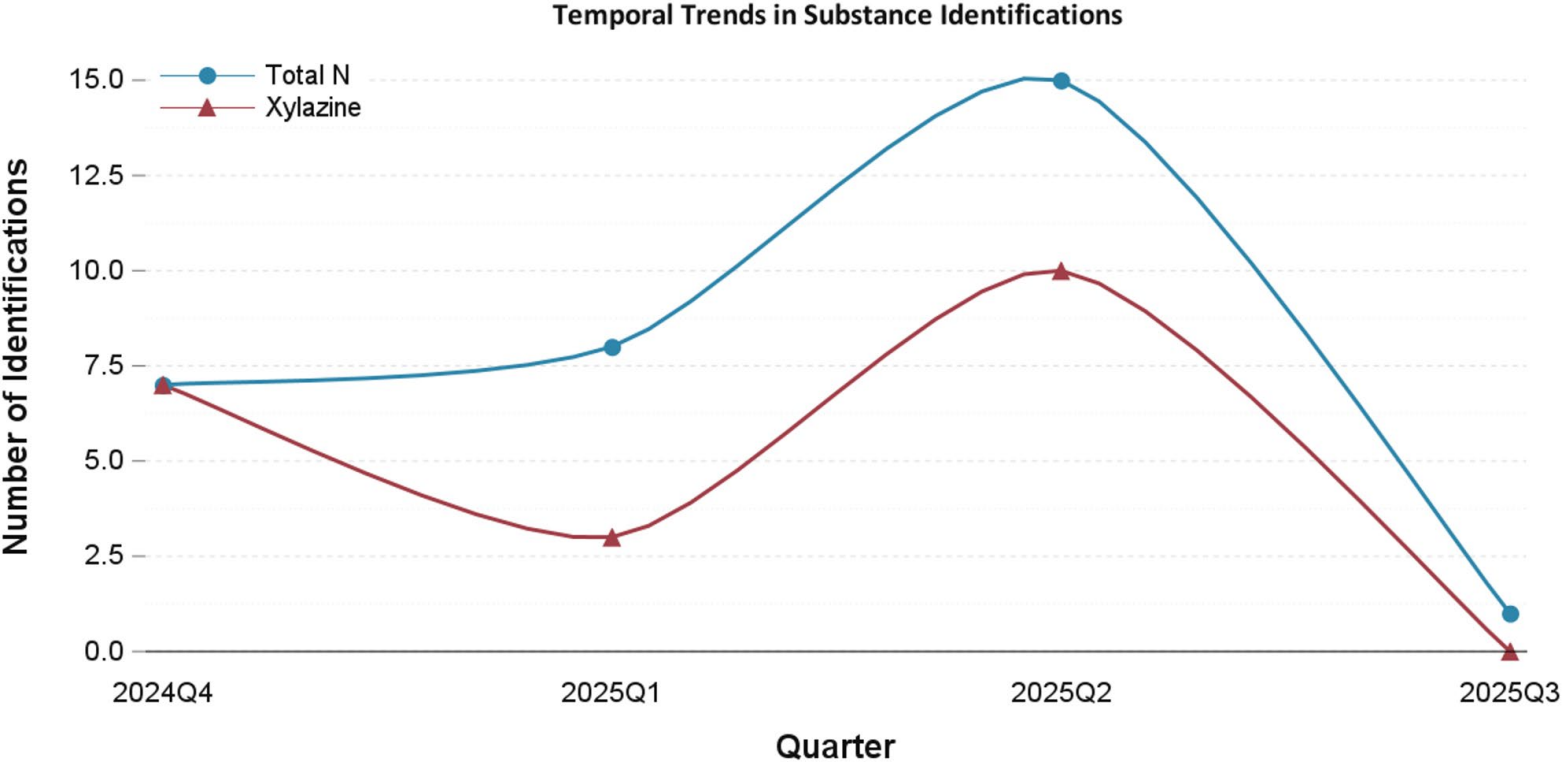
Xylazine identifications by quarter and year

Xylazine was consistently present throughout the study period but did vary slightly in prevalence over time (Fig. 2). The most common co-occurring substances were methamphetamine (*n* = 27, 87.1%) and cocaine (*n* = 14, 45.2%). Buprenorphine and/or methadone was identified in 11 (35.5%) participants. Polysubstance use was common overall, with a mean of 3.7 classes of substances detected (standard deviation [SD] 1.2). Outside of fentanyl, MOUD were the most identified opioids, with buprenorphine (*n* = 7, 22.6%) and methadone (*n* = 4, 12.9%) being most common. Heroin (*n* = 2, 6.5%), hydrocodone (*n* = 1, 3.2%), and oxycodone (*n* = 1, 3.2%) were relatively rare. Substances typically used as cutting agents (lidocaine, levamisole, quinine, caffeine) were very common, with at least one such substance identified in 28 (90.3%) cases. Among these specific agents, the most identified was caffeine (*n* = 23, 74.2%), followed by lidocaine (*n* = 17, 54.8%), quinine (*n* = 14, 45.2%), and levamisole (*n* = 2, 6.5%).

Other detected substances of interest included one detection of medetomidine in May 2025, one detection of desalkylgidazepam, four (12.9%) detections of methcathinone, and frequent identifications of psychiatric medications in 10 (32.3%) persons. Fentanyl analogues (comprised of acetyl fentanyl, fluorofentanyl, *o*-methylfentanyl, para-fentanyl, and para-fluorofentanyl) were detected in a relatively large proportion of participants (*n* = 15, 48.4%), with *o*-methylfentanyl identified in 9 (29.0%) participants. All cases of *o*-methylfentanyl exposure were associated with xylazine co-detection. Only three participants (9.7% of those with toxicology testing) were successfully contacted regarding the results of their xylazine testing.

### Findings by xylazine exposure status

Relative to those who tested negative for xylazine exposure, participants who tested positive tended to be younger (37.6 vs. 42.3 years, *p* = 0.19), positive for fentanyl analogs (56.0 vs. 16.7%, *p* = 0.17), and have more classes of substance detected (3.9 vs. 2.8, *p* = 0.06), although these contrasts did not reach conventional criteria for statistical significance. Cocaine (52.0 vs. 16.7%, *p* = 0.18) and *o*-methylfentanyl (36.0 vs. 0.0%, *p* = 0.14) had the strongest trends toward association with xylazine exposure among individual substances evaluated. Exposed versus nonexposed persons had similar rates of homelessness (48.0 vs. 66.7%, *p* = 0.66), wounds (40.0 vs. 50.0%, *p* = 0.68), anemia (32.0 vs. 50.0%, *p* = 0.64), dysglycemia (8.0 vs. 16.7%, *p* = 0.49), and DVT/PE (12.0 vs. 0.0%, *p* = 1.00). The estimated prevalence of Hepatitis C virus (HCV) diagnosis was higher in participants with xylazine (60.0 vs. 33.3%, *p* = 0.37) although this too fell short of statistical significance. Risky injection behaviors were common across both exposure groups (76.0 vs. 100.0%, *p* value not applicable). Participants who tested positive for xylazine tended to be more likely to have heard of it (60.0 vs. 16.7%, *p* = 0.13) and to report use of xylazine (48.0 vs. 16.7%, *p* = 0.36). Both groups had a strong preference to avoid xylazine (76.0 vs. 83.3%, *p* = 1.00).

### Findings by reported xylazine use

Using the alternative exposure definition of participant-reported xylazine use (Supplemental Table 1), a total of 37 participants were included for primary analysis, with 15 (40.5%) reporting xylazine exposure and 22 (59.5%) reporting no exposure. Of those reporting xylazine exposure, 12 (92.3%) had evidence of exposure by LC-QTOF-MS; of those reporting no exposure, 13 (72.2%) had evidence of exposure (*p* = 0.36). In the total cohort, the typical participant was male (19, 51.4%), white (31, 86.1%), homeless (*n* = 20, 55.6%), rural (*n* = 17, 51.5%), and uninsured (*n* = 13, 41.9%) with an average age of 38.2 (SD 9.8). There were significant between-group differences in homelessness (11 [78.6%] in exposed versus 9 [40.9%] in unexposed, *p* = 0.03), wounds (9 [60.0%] in exposed versus 6 [27.3%] in non-exposed, *p* = 0.046), and fentanyl analog detection (9 [60.0%] in exposed versus 6 [27.3%] in unexposed, *p* = 0.046). There were no statistically significant differences in any of the other xylazine-related comorbidities, use behaviors, or toxicologic entities of interest, and trends were otherwise generally similar to the LC-QTOF-MS testing-based exposure definition.

## Discussion

This pilot cohort study of 37 PWUD demonstrated that ED-based NPS surveillance was feasible and provided novel toxicovigilance information. It reinforces existing work indicating that xylazine exposure is common in people using fentanyl in Alabama, with a point prevalence comparable to those published in similar studies from known high prevalence areas like the Northeast U.S [2, 6, 29]. Within the power confines of a small pilot study, the characteristics of PWUD exposed to xylazine appeared similar to national samples, with polysubstance use, fentanyl analog co-detection, stimulant co-use, and white race all trending toward associations with xylazine exposure [4, 11]. An exposure definition based on laboratory testing found no link between xylazine and wounds or homelessness, but self-reported xylazine use showed the expected associations seen nationally. While limited by a small sample size, this interesting discrepancy raises the possibility that these traditional associations may in fact result from differences in xylazine awareness or participant recall bias rather than reflecting true exposure; this hypothesis-generating finding deserves more study in future, larger studies [11]. Finally, most surveyed PWUD were favorable to improved MOUD and naloxone access. This may reflect the challenging MOUD and harm reduction access landscape in the American Deep South, where lack of Medicaid expansion and lack of rural access to clinics willing to prescribe MOUD greatly hinder access [35, 36]. 

Identification of the novel synthetic opioid *o*-methylfentanyl was unexpected; to our knowledge, this was the first published identification of this drug in Alabama [37]. The fact that this was 100% co-detected with xylazine is notable and suggests that common distribution routes may be shared between *o*-methylfentanyl and xylazine. The identification of medetomidine was also significant in that our study represented the first published identification of this emerging NPS in Alabama [38]. Available data suggest that medetomidine acts similarly to xylazine, with risk of profound sedation and bradycardia [38]. These identifications point to the value of this type of epidemiologic surveillance. Both identifications were presented to state addiction advocacy and treatment organizations who anecdotally reported finding the information valuable in providing services to their clients.

More broadly, our study demonstrates the feasibility of using a residual biological specimen-based screening approach to identify dangerous NPS and drug additives. In Alabama and other states where drug checking is currently illegal, granular and real-time data on substance use behaviors and exposures are sorely needed. In this study, we used an ED-based residual biological specimen testing approach. However, this approach could equally be used to collect low-cost, low-barrier information using residual biological specimens from a diverse array of sources to create a comprehensive and high-quality surveillance network. For example, a state might combine coroner/medical examiner data, ED specimens, and substance treatment clinic specimens with a high throughput LC-QTOF-MS core to create a comprehensive surveillance network for NPS and illicit drug trend tracking. Such a network could allow for timely alerts to PWUD and other important stakeholders in opioid response including public health authorities, healthcare providers, and law enforcement. With new NPS emerging every week, such public health infrastructure is vital to identifying emerging threats in the drug supply [1]. 

Our study is limited in its ability to draw meaningful, generalizable conclusions about xylazine epidemiology, given its small size and single site nature. The small sample size (primarily limited by pilot-level funding) precluded regression analysis. While the UAB ED setting is unique in that it does draw patients from across the state of Alabama (evidenced by the geographic diversity of our sample), this study is not generalizable to rural counties, as the ED is based in an urban location. Additionally, our survey-based methodology involving in person administration of a survey on a highly stigmatized topic, while feasible and inexpensive, may introduce self-report bias. Audio computer-assisted self-interviewing (ACASI) is widely recognized as best practice in this field [39]. While we did take some steps in our survey administration technique to reduce social desirability bias (ensuring private survey administration areas, having the research assistant leave the room during completion), this may have been reflected in survey responses. Our limited, convenience-based sampling (Monday through Friday business hours) may also have introduced recruitment bias. We did have a number of missing specimens primarily because of issues with requisitioning the samples prior to the discard time; this was successfully addressed by the end of the pilot period but led to losing samples early in the study. Finally, the prolonged laboratory turnaround time (median of 55 days) was long and limited our ability to perform timely surveillance and inform participants of their results. In-house high throughput testing would obviate this need.

A more comprehensive sampling strategy focused on rural counties would be an important next step in this crucial line of work. Rural individuals are among the most impacted by the opioid epidemic but remain heavily underrepresented in research due in large part to logistical barriers that make sampling challenging [35, 36, 40]. Separately, an expanded project should make use of best practices in data collection around stigmatized topics [39]. A larger methodological problem in this type of analysis is the unknown pharmacokinetic profiles of many NPS and drug additives. Some compounds with very short half-lives may not be suitable for surveillance in a manner that requires stability for at least a period of hours while the residual biological specimen is stored in the lab and transported to the LC-QTOF-MS facility. This is a limitation that is unique to residual biological specimen-based approaches and not to conventional drug checking.

## Conclusion

We present here a feasible residual biological specimen-based approach to drug surveillance that produces actionable, individual-level information related to NPS and other substance use trends. This approach is scalable, legally viable even in restrictive implementation environments, and well-suited for broader deployment. Future studies should explore implementing this approach on a state or region-wide scale to produce robust, population-level insights into emerging drug use and NPS exposure patterns.

## Supplementary Information

Below is the link to the electronic supplementary material.

Supplementary Material 1

## Data Availability

The datasets used and/or analyzed during the current study are available from the corresponding author on reasonable request.

## Abbreviations

ACASI: Audio computer-assisted self interviewing
CFSRE: Center for Forensic Science Research and Education
DVT: Deep vein thrombosis
ED: Emergency department
EHR: Electronic health record
HCV: Hepatitis C virus
HIV: Human immunodeficiency virus
IRB: Institutional review board
IDU: Injection drug use
LC-QTOF-MS: Liquid chromatography-quadrupole time of flight mass spectrometry
MOUD: Medications for opioid use disorder
NPS: Novel psychoactive substances
PE: Pulmonary embolism
PWUD: People who use drugs
SD: Standard deviation
SSP: Syringe service program
UAB: University of Alabama at Birmingham
UDS: Urine drug screen
US: United States
