# Supplementary material for: Emergency department-based testing for xylazine and other novel psychoactive substances in Central Alabama: a feasibility study
Source: Harm Reduct J. 2026 Jan 17;23:31. doi: 10.1186/s12954-026-01401-5 (PMC12895971; doi:10.1186/s12954-026-01401-5)
Supplement: Supplementary file 1 — Supplementary Material 1 [file 12954_2026_1401_MOESM1_ESM.docx]

**Supplemental Materials**

**Table S1.** Participant characteristics, comorbidities, use behaviors, outcomes, toxicology results, and xylazine knowledge by self-reported xylazine use (N=37).

| **Characteristic** | **Total (N = 37)** | **Self-reported xylazine use (n = 15)** | **No self-reported xylazine use (n =22)** | ***p*-value** |
| --- | --- | --- | --- | --- |
| **Sociodemographics** |  |  |  |  |
| Age (mean [SD]) | 38.2 (9.8) | 35.1 (8.5) | 40.4 (10.2) | 0.16 |
| Sex, n (%) |  |  |  | 0.84 |
| Male | 19 (51.4) | 8 (53.3) | 11 (50.0) |  |
| Female | 18 (48.6) | 7 (46.7) | 11 (60.0) |  |
| Race, n (%) |  |  |  | 0.38 |
| White | 31 (86.1) | 14 (93.3) | 17 (81.0) |  |
| Black/African American | 5 (13.9) | 1 (6.7) | 4 (19.0) |  |
| Missing | 1 | 0 | 1 |  |
| Homeless, n (%) | 20 (55.6) | 11 (78.6) | 9 (40.9) | **0.03** |
| Missing | 1 | 1 | 0 |  |
| Insurance type, n (%) |  |  |  |  |
| Private | 12 (38.7) | 4 (33.3) | 8 (42.1) | 0.72 |
| Public | 6 (19.4) | 1 (8.3) | 5 (26.3) | 0.36 |
| Uninsured | 13 (41.9) | 7 (58.3) | 6 (31.6) | 0.14 |
| Missing | 6 | 3 | 3 |  |
| Rural, n (%) | 17 (51.5) | 9 (69.2) | 8 (40.0) | 0.10 |
| Missing | 4 | 2 | 2 |  |
| Miles from Home to ED (Median [Range]) | 20.6 (1.1-201.3) | 29.2 (4.3-201.3) | 14.7 (1.1-88.9) | 0.32 |
| **Medical History** |  |  |  |  |
| Past year history, n (%) |  |  |  |  |
| DVT/PE | 3 (8.1) | 1 (6.7) | 2 (9.1) | 1.00 |
| Anemia | 15 (40.5) | 8 (53.3) | 7 (31.8) | 0.19 |
| Dysglycemia | 4 (10.8) | 2 (13.3) | 2 (9.1) | 1.00 |
| Compartment syndrome or rhabdomyolysis | 1 (2.7) | 1 (6.7) | 0 (0.0) | 0.41 |
| Wounds | 15 (40.5) | 9 (60.0) | 6 (27.3) | **0.047** |
| Ever had xylazine-associated wounds, n (%) | 9 (30.0) | 7 (46.7) | 2 (13.3) | **0.046** |
| HCV diagnosis, n (%) | 20 (54.1) | 7 (46.7) | 13 (59.1) | 0.46 |
| HCV treatment, n (%) | 3 (15.8) | 2 (28.6) | 1 (8.3) | 0.52 |
| Missing | 18 | 8 | 10 |  |
| HIV diagnosis, n (%) | 2 (5.4) | 0 (0.0) | 2 (9.1) | 0.50 |
| **Medical Outcomes** |  |  |  |  |
| 3-month readmission, n (%) | 1 (3.1) | 1 (7.7) | 0 (0.0) | 0.41 |
| 3-month mortality, n (%) | 0 (0.0) | 0 (0.0) | 0 (0.0) | N/A |
| **Use behaviors** |  |  |  |  |
| IDU, n (%) |  |  |  |  |
| Ever | 30 (81.1) | 12 (80.0) | 18 (81.1) | 0.89 |
| Current | 25 (67.6) | 11 (73.3) | 14 (63.6) | 0.54 |
| Intentional stimulant coinjection, n (%) | 15 (57.7) | 8 (66.7) | 7 (50.0) | 0.39 |
| Missing | 11 | 3 | 8 |  |
| Any IDU-related risk behavior, n (%) | 30 (100.0) | 12 (100.0) | 18 (100.0) | N/A |
| Missing | 7 | 3 | 4 |  |
| Self-reported typical opioid use, n (%) |  |  |  |  |
| Fentanyl | 36 (97.3) | 15 (100.0) | 21 (95.5) | 1.00 |
| Heroin | 17 (45.9) | 6 (40.0) | 11 (50.0) | 0.55 |
| Prescription pills | 5 (13.5) | 3 (20.0) | 2 (9.1) | 0.38 |
| Non-prescription pills | 8 (21.6) | 3 (20.0) | 5 (22.7) | 1.00 |
| **Xylazine understanding** |  |  |  |  |
| Heard of xylazine, n (%) | 20 (62.5) | 15 (100.0) | 5 (29.4) | <0.001 |
| Missing | 5 | 0 | 5 |  |
| Xylazine use preference, n (%) |  |  |  | 0.97 |
| Very much prefer to avoid | 27 (73.0) | 11 (73.3) | 16 (72.7) |  |
| Any other answer | 10 (27.0) | 4 (26.7) | 6 (27.3) |  |
| **Toxicology results** |  |  |  |  |
| Drug class detection, n (%) |  |  |  |  |
| Barbiturates | 1 (2.7) | 0 (0.0) | 1 (4.5) | 1.00 |
| Benzodiazepines | 11 (29.7) | 4 (26.7) | 7 (31.8) | 0.74 |
| Buprenorphine | 10 (27.0) | 3 (20.0) | 7 (31.8) | 0.48 |
| Cocaine | 16 (43.2) | 7 (46.7) | 9 (40.9) | 0.73 |
| Fentanyl | 37 (100.0) | 15 (100.0) | 22 (100.0) | N/A |
| Hydrocodone | 1 (2.7) | 0 (0.0) | 1 (4.5) | 1.00 |
| Methadone | 5 (13.5) | 3 (20.0) | 2 (9.1) | 0.38 |
| Methamphetamine | 31 (83.8) | 13 (86.7) | 18 (81.8) | 0.69 |
| Oxycodone | 1 (2.7) | 0 (0.0) | 1 (4.5) | 1.00 |
| Any MOUD agent | 15 (40.5) | 6 (40.0) | 9 (40.9) | 0.96 |
| Number of Classes of Substance Detected (Mean [SD]) | 3.7 (1.2) | 3.6 (1.1) | 3.7 (1.2) | 0.66 |
| LC-QTOF-MS Detection, n (%) |  |  |  |  |
| Cutting agents | 28 (75.7) | 12 (80.0) | 16 (72.7) | 0.61 |
| Fentalogs | 15 (40.5) | 9 (60.0) | 6 (27.3) | **0.046** |
| Psychiatric medications | 10 (27.0) | 5 (33.3) | 5 (22.7) | 0.48 |
| Methcathinone | 4 (12.9) | 2 (15.4) | 2 (11.1) | 1.00 |
| *O*-methylfentanyl | 9 (29.0) | 5 (38.5) | 4 (22.2) | 0.33 |
| Xylazine | 25 (80.6) | 12 (92.3) | 13 (72.2) | 0.36 |
| Missing | 6 | 2 | 4 |  |

Abbreviations: DVT: Deep vein thrombosis; ED: Emergency department; HCV: Hepatitis C virus; HIV; Human immunodeficiency virus; IDU: Injection drug use; LC-QTOF-MS: Liquid chromatography-quadrupole time of flight mass spectrometry; PE: Pulmonary embolism; SD: Standard deviation
